# Retrospective observational RT-PCR analyses on 688 babies born to 843 SARS-CoV-2 positive mothers, placental analyses and diagnostic analyses limitations suggest vertical transmission is possible

**DOI:** 10.52054/FVVO.13.1.001

**Published:** 2021-03-31

**Authors:** G Bahadur, M Bhat, S Acharya, D Janga, B Cambell, J Huirne, W Yoong, A Govind, J Pardo, R Homburg

**Affiliations:** Reproductive Medicine Unit/Obstetrics and Gynaecology Unit, North Middlesex University Hospital, Old Admin Block, Sterling Way, London N18 1QX, UK; Homerton Fertility Unit, Homerton University Hospital, Homerton Row, London E9 6SR,UK; Ayrshire Fertility Unit, University Hospital Crosshouse, Kilmarnock, Scotland; University Medical Centers Amsterdam, Research Institute Reproduction and Development. Amsterdam, The Netherlands

**Keywords:** COVID-19, SARS-CoV-2, pregnancy, risks, vertical transmission

## Abstract

**Research question:**

Is there vertical transmission (from mother to baby antenatally or intrapartum) after SARS-CoV-2 (COVID-19) infected pregnancy?

**Study design:**

A systematic search related to SARS-CoV-2 (COVID-19), pregnancy, neonatal complications, viral and vertical transmission. The duration was from December 2019 to May 2020.

**Results:**

A total of 84 studies with 862 COVID positive women were included. Two studies had ongoing pregnancies while 82 studies included 705 babies, 1 miscarriage and 1 medical termination of pregnancy (MTOP). Most publications (50/84, 59.5%), reported small numbers (<5) of positive babies. From 75 studies, 18 babies were COVID-19 positive. The first reverse transcription polymerase chain reaction (RT-PCR) diagnostic test was done in 449 babies and 2 losses, 2nd RT-PCR was done in 82 babies, IgM tests were done in 28 babies, and IgG tests were done in 28 babies. On the first RT-PCR, 47 studies reported time of testing while 28 studies did not. Positive results in the first RT-PCR were seen in 14 babies. Earliest tested at birth and the average time of the result was 22 hours. Three babies with negative first RT-PCR became positive on the second RT-PCR at day 6, day 7 and at 24 hours which continued to be positive at 1 week.

Four studies with a total of 4 placental swabs were positive demonstrating SARS-CoV-2 localised in the placenta. In 2 studies, 10 tests for amniotic fluid were positive for SARS-CoV-2. These 2 babies were found to be positive on RT-PCR on serial testing.

**Conclusion:**

Diagnostic testing combined with incubation period and placental pathology indicate a strong likelihood that intrapartum vertical transmission of SARS-CoV-2 (COVID-19) from mother to baby is possible.

## Introduction

In the current SARS-CoV-2 (COVID-19) pandemic, testing has become a cornerstone for public health advice, policies and political agenda. The unprecedented and exponential growth of non-peer- reviewed publications, systematic reviews lacking rigorous thoroughness and the repetitive inclusion of case reports is problematic (Bauchner, 2020). We critically appraised the limitations and manner of diagnostic testing in obstetric medicine (Bahadur et al., 2020). While the genetic sequence for SARS- CoV-2 became available (Wang et al., 2020), it was only after mid-April 2020 that profound concerns were aired about the very high false-negative rates for antibodies. This false-negative rate for antibodies has come down from almost 1 in 2 to 1 in 3 tests, possibly because the reverse transcription polymerase chain reaction (RT-PCR) tests were based on the previous SARS family virus (Bahadur et al., 2020).

The second major limitation is the fact that most researchers had not taken account of the incubation period, which for SARS-CoV-2 is 5-6 days (Li Q et al., 2020) and most publications have relied on a single test rather than serial checks, thereby producing scientifically unreliable conclusions. There is also the possibility that latent infections exist even before the onset of symptoms (Anderson et al., 2020) and the earliest trigger for transmission occurs before the onset of symptoms which could be one to two days if with viraemia (Zou et al., 2020). The safety margins to assess the likelihood of infection before the onset of infection should be revised towards 7-8 days, which is different from the World Health Organisation (WHO) RT-PCR testing recommendation for the asymptomatic or mildly symptomatic (WHO, 2020). Asymptomatic carriers acquiring and transmitting SARS-CoV-2 remains an underexplored area (Bai et al., 2020) against those cases with confirmed SARS- CoV-2 clinical symptoms (Huang et al., 2020).

Serological testing is a measure of a by-gone event (WHO, 2020) with probable clues on the long-term immunity or immunological memory and route to immunisation (Dijkstra et al., 2020). IgM provides the initial defence to viral infections, before generating the adaptive IgG response. From 173 SARS-CoV-2 infected patients and 535 plasma samples, the median seroconversion time for total antibody (Ab), IgM and then IgG was day 11, day 12 and day 14, respectively. The antibodies were present in <40% among patients within 1-week of onset, and rapidly increased to 100 % (Ab), 94.3% (IgM) and 79.8% (IgG) 15 days after onset (Jacofsky et al., 2020; Li Z et al., 2020). Furthermore, cross-reactivity against multiple coronavirus strains with similar antigen sites can become problematic (Jacofsky et al., 2020; Xiao et al., 2020).

This retrospective observational study on RT-PCR analyses on babies born to SARS-CoV-2 positive mothers sets out to analyse how SARS-CoV-2 diagnostic analyses and placental analyses were performed, their scientific limitations in interpreting data and whether intrapartum vertical transmission from mother to baby was possible.

## Methods

### Study design, size, duration

A systematic review searching terms related to SARS-CoV-2, COVID-19, pregnancy, neonatal complications, viral and vertical transmission was conducted. No language restriction was placed on published articles. The duration was from 1st December 2019 to 15th May 2020 for extracting the literature and screening the articles of potential interest.

### Participants/materials, setting, methods

A systematic review following the PRISMA format was performed in PUBMED, EMBASE, CENTRAL, WEB of SCIENCE, Web of Knowledge, the WHO, RCOG, ESHRE, ASRM, NEJM, BMJ, Lancet, Welcome and Cochrane Central Register of Studies, UKOSS, Office of National Statistics (ONS-UK), Department of Health (UK), Google Scholar and any references of relevant articles. Data relevant to SARS-CoV-2 diagnostic interpretation was also collected. Case reports or case series of pregnant women with confirmed COVID-19, where neonatal outcomes were reported were assembled on an Excel spreadsheet.

## Results

A total of 84 studies with 862 COVID-19 positives were included. Two studies had ongoing pregnancies while the remaining 82 studies included 705 babies, one miscarriage and one medical termination of pregnancy (MTOP).

**Figure 1:**
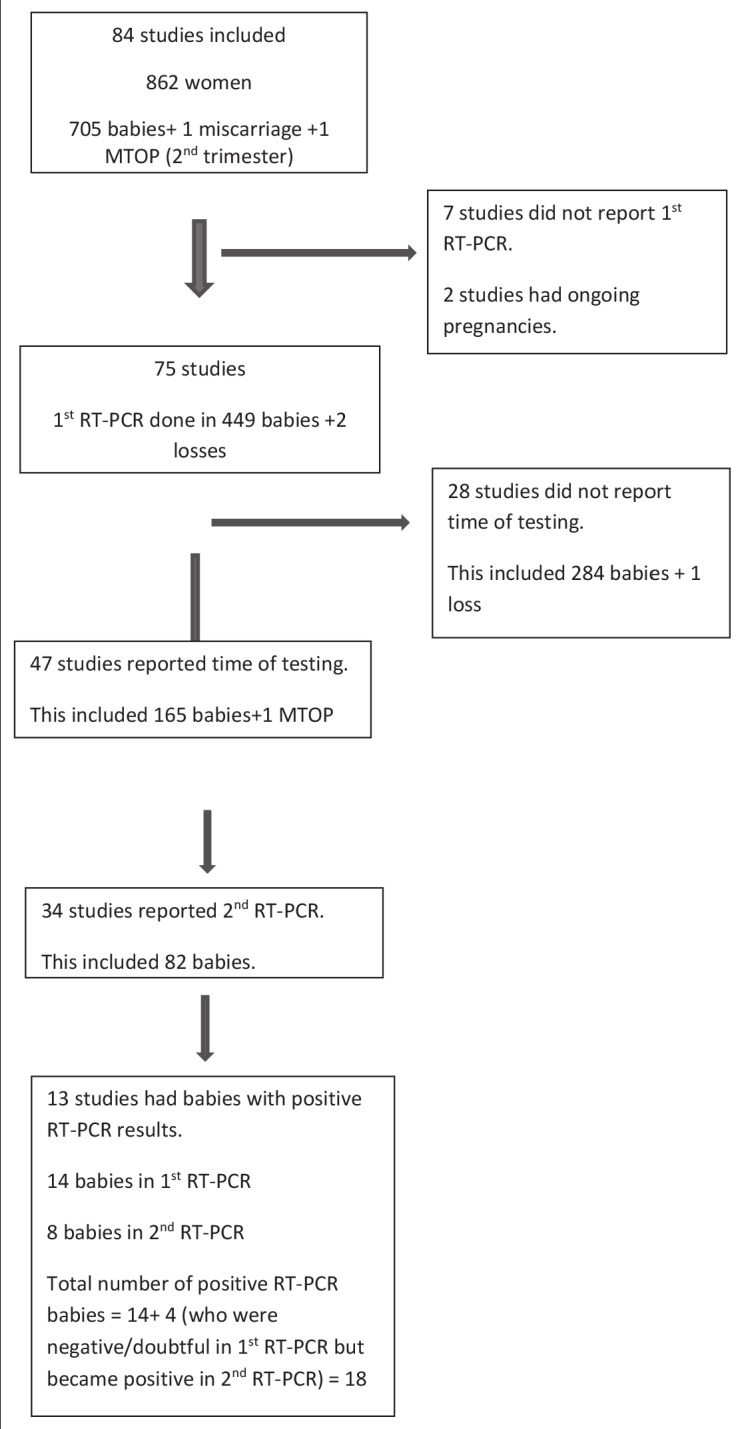
— 1^st^ and 2^nd^ RT PCR testing in babies born to COVID 19 positive mothers.

Of the 84 studies, 1st RT-PCR was undertaken in 449 babies and two losses in 75 studies. Seven studies did not report testing, while two had ongoing pregnancies. Forty-one studies had only one RT- PCR testing done. 34 studies had a 2nd RT-PCR done. Overall excluding two losses, there were 431 negative, 14 positive and four unclear results (total=449) in the 1st RT-PCR. 356/431(82.5%) babies had only one negative RT-PCR (no serial testing). The time at which 1st RT-PCR was done was recorded in 47 studies (165/449 babies) but not in 28 studies (284/449 babies). Considering the 47 studies with 165 babies, the mean times of negative and positive results were 28 hours (day 1 after birth, day 1) and 22 hours (day of birth, day 0) respectively. 2nd RT-PCR was reported in 34 studies and 82 babies. 74 were negative and eight were positive. The mean time of negative result was 92 hours / day 4 (Figure 1).

Positive results in 1st RT-PCR were documented in 14 babies. The earliest were tested at birth and the average time of the result was 22 hours/ day 0. Three babies with negative 1st RT-PCR became positive in 2nd RT-PCR at D6, D7 and at 24 hours which continued to test positive at 1 week. One baby with an equivocal result with 1st RT-PCR became positive in subsequent testing on day 3 (Figure 1, Table 1).

**Table I:** RT-PCR results in babies tested positive.

Study	No. of babies tested/total no. of babies in the study	1^st^ RT-PCR	Time at which 1^st^ RT-PCR done	2^nd^ RT-PCR	Time at which 2^nd^ RT-PCR done	3^rd^ and subsequent RT-PCR	Time at which test done
Yu N et al	3/7	1 positive	36 hours	1 negative	within 2 weeks	1 negative	Within 2 weeks
Zeng L et al	33/33	3 positive	Day 2 & 4	3 negative	2 on Day 6 & 1 on day 7		
Wang S et al	1/1	1 positive	36 hours	1 negative	Day 15		
Ferrazzi E et al	42/42	2 positive 1 doubtful	Day 1	2 positive 1 positive	Day 3		
Alzamora MC et al	1/1	1 positive	16 hrs	1 positive	48 hrs		
Khan S et al	17/17	2 positive	24 hrs	Not tested			
Nie R et al	26/28	1 positive	36 hrs	1 negative	Day 4	1 negative	Day 8 & 15
Carosso A et al	1/1	1 positive	0 hr	1 negative	37 hrs		
Hu X et al	7/7	1 positive	36 hr	Not tested			
Diaz SA et al	1/1	1 negative	Day 6	1 positive	Day 8	1 positive	Day 13
Zamaniyan M et al	1/1	1 negative	0 hr	1 positive	24 hrs	1 positive	1 week
Hantoushzadeh S et al	5/9	5 negative	Day 1	1 positive	Day 7		
Kirtsman M et al	1/1	1 positive	At birth	1 positive	Day 2	1 positive	Day 3

This table includes all babies who were positive on RT-PCR at some point. Out of the 18 babies who were positive at some point, only 3 have positive result after 5 days of life. These 3 babies had initial negative result. All the rest had positive result within first 5 days and either became negative or were not tested further or continued to have positive result but these were within first 5 days.

Antibody testing (IgM/IgG) was done in 28 babies from six studies. Twenty-one babies had negative results, while 7 (25%) were positive (3 had positive IgM and all 7 had positive IgG). All three babies with positive IgM had negative RT-PCR. IgG could be acquired by transfer from mum through the placenta. All except one baby with positive IgG had a positive 1st RT-PCR. When this same swab was repeated after 37 hours, RT-PCR was negative. In the two studies where babies were positive for IgM, no information was available on placental swabs, cord blood or amniotic fluid.

One baby with negative antibody testing had positive 1st RT-PCR at 16 hrs after birth and the mother became antibody positive on postpartum day 4.

In order to confirm vertical transmission, amniotic fluid, and placenta and cord blood were assessed for evidence of infection with SARS-CoV-2 by RT-PCR results. In 15 studies (46 women) had sent placental swabs. Four studies with a total of four placental swabs were positive. Positive placental swabs on histology demonstrated SARS-CoV-2 localised predominantly to the syncytiotrophoblast cells of the placenta.

In 17 studies, amniotic fluid from 67 women was tested for COVID-19. In two studies, 10 tests were positive. In these, two babies were found to be positive on RT-PCR on serial testing.

Cord blood samples were tested in 77 cases (in 15 studies) and all except one was negative. This particular study included one woman who had a MTOP for maternal compromise with severe pre- eclampsia and COVID-19 infection with possible placental abruption. Fetal heart and lung tissues tested negative for SARS-CoV-2, whilst placenta and cord blood were found to be positive.

Out of the 705 babies and 2 non-viable fetuses, 18 babies were found to be positive on at least one RT-PCR testing. Timing and serial testing were done in 82 babies (>= 2 tests).

## Discussion

Pregnant women are considered to be a high-risk group for SARS-CoV-2 infection, with potentially adverse maternal and perinatal outcomes, with an increased risk of admission to an intensive care unit (ICU) (Allotey et al., 2020). There was an increased likelihood of admission to the ICU (odds ratio (OR) 1.62, 95% CI 1.33 - 1.96) and requiring invasive ventilation (OR 1.88, 95% CI 1.36 -2.60) among pregnant women compared to non-pregnant women with COVID-19. Confirmed SARS-CoV-2 positive patients had a higher risk of delivering preterm and infants born to these mothers were more likely to be admitted to the neonatal intensive care unit. A quarter of all neonates from positive mothers were admitted to ICU. There appeared to be a 7% positivity rate among women universally screened for COVID-19 during pregnancy (Allotey et al., 2020). The rates of spontaneous and overall preterm birth were 6% and 17% respectively, while of the 11,000 pregnant women with SARS-CoV-2, 73 died (Allotey et al., 2020). Of 598 hospitalised pregnant women with SARS-Cov-2, about 55% were asymptomatic (Delahoy et al., 2020). Vertical transmission and congenital abnormalities experienced with Zika virus raise concerns about SARS-Cov-V-2 (Krauer et al., 2017; Hintley et al., 2020).

**Table II:** Maternal characteristics.

Study	Age	Parity	Gestation	Contact history	Co-morbidities	Outcome
Yu N et al	38	G3P2	39+6	No	Hypothyroidism	recovered
Zeng L et al			40	NR ^x^	NR	recovered
			40+4	NR	NR	recovered
			31+2	NR	NR	recovered
Wang S et al	34		40	Yes	Hypothyroidism	recovered
Ferrazzi E et al	Mean-34.6			NR	NR	recovered
Alzamora MC et al	41	G3P2	33	Yes	Prev 2 CS Diabetes	recovered
Khan S et al	29(24-34)		38(35+5 -41)	Yes	NR	recovered
Nie R et al				NR	NR	recovered
Carosso A et al	28	G2P1	37	NR	GDM	recovered
Hu X et al	34		40	Yes	None	recovered
Diaz SA et al	41		38+4	Yes	Hypothyroidism, IVF pregnancy, severe PET	ventilated
Zamaniyan M et al			32+2	Yes	hypothyroidism	Died
Hantoushzadeh S et al	40-44	G2P1	30+5	No	Subclinical hypothyroidism, advanced maternal age	Died
Kirtsman M et al	40	G2P1	35+5	NR	familial neutropenia, GDM, recurrent bacterial infection	recovered

Hypothyroidism and advanced maternal age were a common maternal morbidity. Elderly mums. For babies with positive results after D5 – 2 mums were critically ill and died after delivery. ^x^NR- Not recorded.

If more than half of SARS-CoV-2 infected pregnant women were asymptomatic there are healthcare implications in managing all patients and staff within a clinical setting (Delahoy et al., 2020). Equally the meaningfulness of the SARS-CoV-2 diagnostic analyses needs to be addressed against the backdrop of high levels of false negatives results relying on a single test which would thereby not inform us a true value of SARS-CoV-2 vertical transmission (Woloshin et al., 2020). Our data and interpretation suggest the need to improve our understanding when testing has to be performed and when to repeat to reach a meaningful clinical decision process and especially where validation of test had not occurred, the sensitivity not reported and where gold standard and controls remained obscure. What is now known is that there can be 100% false negatives on the first day of showing symptoms from an infected individual and dropping to 67% on day 4. Thus, the validity of the RT-PCR post symptoms would only be valid from days 5 to 12 after which RT-PCR becomes negative (Kucirka et al., 2020). In four cohorts, 100% of patients initially tested negative but turned positive after 2nd and 3rd re-tests for SARS-CoV-2 RNA (Kelly et al., 2020).

False-negative testing for SARS-CoV-2 is a serious clinically relevant problem and this should be overcome by serial testing in the 5-7 days incubation period post-infection (Kelly et al., 2020). In our study cohort there is no evidence that any of the researchers have been aware of the relevance of timing of testing or the need for serial testing to ensure the repeatability of an initial negative result thereby leading to inappropriate reassurances and conclusions about the health and status of SARS- CoV-2 infected mothers and babies with all its implications for healthcare workers in attendance.

The most striking feature from our data analyses of 84 studies was the over-reliance on a single RT- PCR negative test in 41 papers and the absence of documentation of time that the sample were taken in 28 papers (Figure 1). Given the incubation period, a single result cannot be used meaningfully, and conclusions remain unsupported by the available evidence. In 82.5% babies, only 1 negative RT- PCR done on an average on day 1 was relied upon. Even in babies having had 2nd negative RT-PCR, the test was done within 96 hours/ day 4. Based upon the understanding that the incubation period is > 5 days, the validity of this testing is questionable. In the few sequential tests performed these appeared more random rather than scientific reasoning in relation to the incubation period for SARS-CoV-2 viral infection.

Antibody testing has very limited value in unravelling the status of vertical transmission. IgG molecules can travel across the placental membranes; hence its’ presence does not support or refute vertical transmission. In two studies involving babies having positive IgM, there was no other supporting information supporting vertical transmission. In addition, RT-PCR in these babies was negative. From our analyses there remains sparse and meaningless information of the time for the production of IgM and IgG along with limited publication and possibly antibodies are more significant for long term immunity.

The strength of our study was the analysis of a substantial data set of publications and the manner in which the diagnostic tests were performed, revealing poor interpretation and meaningful follow up. Given the diagnostic tests were developed on the SARS Middle Eastern strain and in the period of study, the validation of these tests had not been established or undertaken and this adds to the weaknesses over which we do not have control.

Little information existed in the validation of diagnostic tests being utilised. Numerous tests have come onto the market since these studies were reported with differing sensitivities and specificities (Corman et al., 2020; Bahadur et al., 2020; Laureano and Riboldi, 2020) and its use to derive reassurances on major healthcare implication for mothers, babies and healthcare workers need caution. Single occasion screening is likely to miss potentially infected people (Viswanathan et al., 2020) and clinicians should not rely on unexpected negative results and performing serial tests could overcome an individual test’s limited sensitivity. Recent studies though not covered in our analyses are unlikely to alter the findings of this study as concerns about the lack of serial diagnostic analyses remain. As far as we can ascertain this risk is minimal in our cohort analyses and our study remains robust and contributes to the growing body of information on maternal-fetal effect during the SARS-CoV-2 pandemic.

Vertical transmission from mother to baby is rare and when this occurs there are serious health implications for the baby. The SARS-CoV-2 studies included in our cohort do cover placental pathology reports (Hosier et al., 2020, Kirstman et al., 2020, Schoenmakers et al., 2020, Shanes et al., 2020). The histological analyses in these reports point to a strong indication that intrapartum vertical transmission from mother to fetus had occurred (Table VIII). Our data also highlight the need to look at vertical transmission alongside placental pathologies where SARS-CoV-2 invasion of the placenta shows the potential for severe morbidity in pregnant women (Hosier et al., 2020). Another likely case of vertical transmission indicates neonates should have SARS-CoV-2 testing of the nasopharynx, placenta and cord blood as soon as possible after birth, while sample timing and collection methods should be documented (Kirtsman et al., 2020). There is limited information on how long the women were positive for SARS Cov2 virus and how soon transplacental transmission occurs. Assuming babies acquired infection in utero nearer delivery, it would be expected that the babies would be positive after the incubation period, most likely in the five days following delivery. In our review, there were only two babies who were negative soon after delivery but became positive five days later and one who became positive at 24 hours and continued to be positive at one week. All the other babies had positive RT-PCR within five days of birth. Of these three babies, two had positive amniotic fluid raising the possibility of vertical transmission (Table VI). Had it been contamination from amniotic fluid, RT- PCR would be expected to be positive in the initial testing soon after birth (Table VII).

**Table VI:** Placenta, amniotic fluid, cord blood, breast milk, vaginal swab results.

Study	Placenta	Amniotic fluid	Cord blood	Vagina	Breast milk
Yu N et al	Negative		Negative		
Zeng L et al		Negative	Negative		Negative
		Negative	Negative		Negative
		Negative	Negative		Negative
Wang S et al	Negative		Negative		Negative
Ferrazzi E et al					
					
Alzamora MC et al					
Khan S et al					
Nie R et al	Negative		negative		
Carosso A et al	fetal and maternal side negative				negative
Hu X et al		negative			
Diaz SA et al					
Zamaniyan M et al		Positive	Negative	Negative	
Hantoushzadeh S et al		Positive			
Kirtsman M et al	Positive		Not tested	Positive	Positive

**Table VII:** Evidence for and against vertical transmission - Inconsistencies in the timing and repetition of RT-PCR makes it difficult to come to an inference

Study	1^st^ RT-PCR	Time done	2^nd^ RT-PCR	Time done	For vertical transmission	Against vertical transmission
Yu N et al	1 pos	36 hours	1 neg	Within 2 week	CS, and baby was isolated for 14 days	Placenta and cord blood neg. Contamination- baby showed mild SOB Don’t know exactly when baby became negative
Zeng L et al	1pos	Day 2&4	1 neg	Day 6	Cs, isolation	Cord blood and amniotic fluid neg. Positive in <5 days
	1pos	Day 2&4	1 neg	Day 6	Cs isolation	Cord blood and amniotic fluid neg. Positive in <5 days
	1pos	Day 2&4	1 neg	Day 7	If vag fluid contamination- why amniotic fluid neg?	Cord blood and amniotic fluid neg. Prematurity complication. Vaginal fluid contamination(PPROM) Ex utero infec?
Wang S et al	1 pos	36 hrs	1 neg	Day 15	Long gap between 1^st^ and 2^nd^ RT-PCR- difficult to know if baby was positive within a week (5-7 days)	Placenta and cord blood neg Baby asymptomatic
Ferrazzi E et al	2 pos	Day 1	2 pos	Day 3		Vaginal fluid contamination Likely contamination. RT-PCR not repeated after 5 days. Babies were relatively asymptomatic.
	1 doubtful	Few hours after birth	1 pos	Day 3		-do-
Alzamora MC et al	1 pos	16 hrs	1 pos	48 hrs	CS Diabetes	Baby positive <5 days. Had resp difficulty and needed ventilated but was premature at 33 weeks? Cause is prematurity.
Khan S et al	2 pos	24 hrs	Not tested		CS neonatal pneumonia	Insufficient information- pos at 24 hours and not repeated.
Nie R et al	1 pos	36 hrs	1 neg	Day 4		Neg at D4 Placenta cord blood neg Isolated for 16 days ?contamination
Carosso A et al	1 pos	0 hr	1 neg	37 hrs (on same swab)		Vaginal contamination highly likely as same swab neg after 37 hours. Possibly low titres...
Hu X et al	1 pos	36 hr	Not tested		Positive >day 7 Isolation CS-?vertical	Amniotic fluid pos ?ex-utero inf
Diaz SA et al	1 neg	Day 67	1 pos	Day 8		High likelihood of ex-utero infection from Mum- Mum BF till D6 when baby showed symptoms and was tested.
Zamaniyan M et al	1 neg	0 hr	1 pos	24 hrs		Amniotic fluid positive. If it was amniotic fluid contamination-should have been positive at birth as well. Cord blood was negative so unlikely vertical transmission? infection exutero?
Yu N et al	1 pos	36 hours	1 neg	Within 2 week	CS, and baby was isolated for 14 days	Placenta and cord blood neg. Contamination- baby showed mild SOB Don’t know exactly when baby became negative
Zeng L et al	1pos	Day 2&4	1 neg	Day 6	Cs, isolation	Cord blood and amniotic fluid neg. Positive in <5 days
	1pos	Day 2&4	1 neg	Day 6	Cs isolation	Cord blood and amniotic fluid neg. Positive in <5 days
	1pos	Day 2&4	1 neg	Day 7	If vag fluid contamination- why amniotic fluid neg?	Cord blood and amniotic fluid neg. Prematurity complication. Vaginal fluid contamination(PPROM) Ex utero infec?

*CS-Caesarean section; **PPROM-Prelabour premature rupture of membranes

**Table VIII:** Placental histology.

Study	No. of women	No. of babies	Placental histology
Schoenmakers S et al	1	1 negative	Placenta showed the presence of SARS-CoV-2 particles with generalized inflammation characterized by histiocytic intervillositis with diffuse perivillous fibrin depositions with damage to the syncytiotrophoblasts.The maternal side of the placenta had a viral load of 4.42 log copies /mL, while the fetal side had 7.15 log copies /mL.
Hosier H et al	1	MTOP-negative	The placenta was remarkable for the presence of diffuse perivillous fibrin and an inflammatory infiltrate composed of macrophages as well as T lymphocytes, consistent with histiocytic intervillositis. SARS–CoV-2 localized predominantly to the syncytiotrophoblast cells of the placenta.
Shanes ED	16	15 babies negative 1 miscarriage- not tested	Placentas show increased prevalence of decidual arteriopathy and other features of maternal vascular malperfusion, a pattern of placental injury reflecting abnormalities in oxygenation within the intervillous space associated with adverse perinatal outcomes.
Kirtsman M et al	1	1 positive	Placenta showed multiple areas of infiltration by inflammatory cells and extensive early infarction, largely confined to the intervillous space, consistent with chronic histiocytic intervillositis. There was extensive early necrosis of the syncytiotrophoblast layer.

*MTOP- Medical termination of pregnancy

Given that the majority of pregnant women appear asymptomatic or have nonspecific symptoms (Table III), it was remarkable to have confirmation of SARS-CoV-2 placental damage, detection of SARS-CoV-2 RNA and the presence of whole viral particles on both maternal and fetal aspects of the placenta (Schoenmakers et al., 2020). Placental histological changes (Kirtsman et al., 2020; Shanes et al., 2020) provide important supporting tools to SARS-CoV-2 diagnostic testing when deciphering vertical transmission, especially considering that the majority of pregnant women appear asymptomatic although other infection routes such as through being born vaginally, breastfed or remaining with the infected mother (Walker et al., 2020). Several infected babies within 12 hours suggest an incubation period pre-existed the delivery to suggest vertical transmission (Table V). PCR positive SARS-CoV-2 tests in the amniotic fluid and infant provide important support for vertical transmission in SARS-CoV-2 infected pregnant women (Zamaniyan et al., 2020). A recent report outside our study cohort provides compelling data on vertical transmission and where the RT-PCR viral load was much higher in placental tissue than in amniotic fluid and maternal or neonatal blood (Vivanti et al., 2020) (Table VIII). Recent studies suggest SARS-CoV-2 vertical transmission, the associated chronic placental insufficiency, as well as miscarriage and fetal growth restriction (Gengler et al., 2020), while a systematic analysis shows a 3.3% intrapartum vertical transmission (Raschetti et al., 2020). Strong evidence of transplacental transmission in early pregnancy associated with hydrops fetalis and fetal demise due to SARS-CoV-2 is also reported (Shende et al., 2020).

**Table III:** Maternal symptoms and investigation.

Study	Symptoms	Lymphopenia	Thrombocytopenia	Transaminitis	Others	RT-PCR (all positive)
Yu N et al	Fever	Yes	yes		↑ CRP CT-pneumonia	Throat swab positive, Sputum & nasopharyngeal postpartum neg
Zeng L et al	Fever, pneumonia					Nasopharyngeal
	Cough, pneumonia					Nasopharyngeal
	Pneumonia					Nasopharyngeal
Wang S et al	Fever, Pneumonia	yes			↑ CRP Neutrophilia	Pharyngeal
Ferrazzi E et al	Pneumonia postpartum					Throat postpartum
Alzamora MC et al	malaise, fatigue, fever, SOB				Metabolic acidosis, pancytopenia, raised CRP	Nasopharyngeal
Khan S et al	Fever, SOB, cough	yes		yes		Pharyngeal
Nie R et al	Fever					Throat
Carosso A et al	mild fever, dry persistent cough					Nasopharyngeal swab positive rectal and stool swab postdelivery positive, vaginal swab postdelivery negative
Hu X et al	Fever	yes		Yes		Throat
Diaz SA et al	Postpartum-fever, pneumonia					Test not specified
Zamaniyan M et al	Myalgia, SOB, anorexia, nausea, non-productive cough, fever	yes			↑ CRP CT-pneumonia	Nasal and throat
Hantoushzadeh S et al	Fever, cough	yes			↑ CRP CT-pneumonia	Nasopharyngeal
Kirtsman M et al	myalgia, decreased appetite, fatigue, dry cough, fever	yes			Raised aPTT	Nasopharyngeal

*CRP- C-reactive protein; **CT-Computed tomography; ***SOB- Shortness of breath

**Table IV:** Baby characteristics.

Study	No. of positive babies	Gestation	Mode of delivery	Apgar score @ 1,5,10 min	Birth weight (gm)
Yu N et al	1	39+6	CS	8-9,9-10	3250
Zeng L et al	1	40	CS	NR	3250
	1	40+4	CS	NR	3360
	1	31+2	CS	3,4,5	1580
Wang S et al	1	40	CS	8,9	3205
Ferrazzi E et al	2		SVD	>7 at 5 min	3224
			SVD	NR (Good condition)	NR
Alzamora MC et al	1	33	CS	6,8	2970
Khan S et al	2	38(35+5 -41)	CS	9,10	3104 (mean)
Nie R et al	1		CS	8-10,9-10	NR
Carosso A et al	1	37	SVD in neg pressure room	9,10	3120
Hu X et al	1	40	CS	8,9	3250
Diaz SA et al	1	38+4	CS	7,9	2500
Zamaniyan M et al	1	32+2	CS	8,9	2350
Hantoushzadeh S et al	1	30+5	CS	9,10	2100
Kirtsman M et al	1	35+5	CS	9,9	2930

*Caesarean section; **SVD-Spontaneous vaginal delivery; ***NR- Not recorded

**Table V:** Baby symptoms & treatment details..

Study	Symptoms & signs	Treatment	NNU admission (days)	Isolation	Breast feeding
Yu N et al	mild SOB	not ventilated	14	NR	
Zeng L et al	lethargy,fever, pneumonia	NR	2	NR	
	lethargy,fever, pneumonia vomiting	NR	4	NR	
	resp distress, pneumonia, sepsis, coagulopathy	non-invasive ventilation, anti-biotic, caffiene	11	NR	
Wang S et al	Clinically well, Ct chest showed changes	Not ventilated, monitored	13	Yes	
Ferrazzi E et al	NR	NR	NR	No	Yes
	GI symptoms. Had resp symptoms after 3 days	Ventilated 24 hrs	1	Yes	No
Alzamora MC et al	mild resp difficulty, sporadic cough	Ventilated	NR	yes	No
Khan S et al	Neonatal pneumonia	NR	NR	NR	
Nie R et al	Pulmonary infection	No treatment required	16	Yes	
Carosso A et al	Asymptomatic	Not ventilated	NR	Yes	
Hu X et al	Asymptomatic	NR	NR	Yes	
Diaz SA et al	Respiratory distress	CPAP x2hours	5	Yes (isolated on day 6 when mum positive)	Yes
Zamaniyan M et al	Fever	Not ventilated	NR	Yes	
Hantoushzadeh S et al	pneumonia, lymphopenia, prematurity	Ventilated	NR	Yes	
Kirtsman M et al	hypoglycemia, feeding difficulty, hypothermia	not ventilated, antibiotics glucose	1	Yes	Yes

*NR- Not recorded ; ** CPAP- Continuous Positive Airway Pressure; ***CT- Computed tomography

It seems that the timing of diagnostic tests and interpretation of results are not standardised in an obstetric medicine setting. From our data, it appears there is inconsistency in the tests, timing and repeatability of RT-PCR, adding to the uncertainty to reach credible inferences leading to likely false and inconsistent healthcare reassurances in relation to SARS-CoV-2 infectivity. Importantly, the incubation period and the timing of testing which could account for false-negative results appear to have been overlooked or not factored-in most studies in our cohort. Where repeat testing was performed this was not based on any critical scientific rationale. Despite a large volume of publications being added every month, there remains a fundamental absence of serial testing and over-reliance on a single test that underestimates the true positive cases as highlighted with our study.

The most important question in reproductive and fetal medicine to be answered is whether there is a vertical transmission of SARS-CoV-2 virus from mother to baby and if there is an increased incidence of congenital malformations. This paper reveals that vertical transmission of SARS-CoV-2 based on diagnostic analyses is not accurately detected and it disregards the viral incubation period, placental pathologies and peripheral supporting data, thereby under-reporting mother to baby vertical transmission. In conclusion, the vast body of information generated during this SARS-CoV-2 pandemic may falsely reassure the public about the overall level of mother to baby viral transmission since serial testing after a negative test was not performed in over 80% of newborns. It is more important to amalgamate the diagnostic testing with good microscopic and histological analyses of the placenta for vertical transmission of SARS-CoV-2 to be assessed. The collective information of diagnostic analysis, incubation periods, placental and amniotic fluid data and increasingly positive cases suggest the argument for intrapartum vertical transmission is compelling and transmission from mother to baby cannot be dismissed.

**Table IX:** List of studies with babies positive for COVID 19.

Study	Year	Title	Link
Yu N et al	2020	Clinical features and obstetric and neonatal outcomes of pregnant patients with COVID-19 in Wuhan, China: a retrospective, single-centre, descriptive study.	https://www.thelancet.com/journals/laninf/article/PIIS1473-3099(20)30176-6/fulltext
Zeng L et al	2020	Neonatal Early-Onset Infection With SARS-CoV-2 in 33 Neonates Born to Mothers With COVID-19 in Wuhan, China.	https://jamanetwork.com/journals/jamapediatrics/fullarticle/2763787
Wang S et al	2020	A Case Report of Neonatal 2019 Coronavirus Disease in China.	https://academic.oup.com/cid/advance-article/doi/10.1093/cid/ciaa225/5803274
Ferrazzi E et al	2020	Mode of Delivery and Clinical Findings in COVID-19 Infected Pregnant Women in Northern Italy.	https://papers.ssrn.com/sol3/papers.cfm?abstract_id=3562464
Alzamora MC et al	2020	Severe COVID-19 during Pregnancy and Possible Vertical Transmission.	https://www.thieme-connect.com/products/ejournals/pdf/10.1055/s-0040-1710050.pdf
Khan S et al	2020	Association of COVID-19 infection with pregnancy outcomes in healthcare workers and general women.	https://www.ncbi.nlm.nih.gov/pmc/articles/PMC7141623/pdf/main.pdf
Nie R et al	2020	Clinical features and the maternal and neonatal outcomes of pregnant women with coronavirus disease 2019.	https://www.medrxiv.org/content/10.1101/2020.03.22.20041061v1.full.pdf
Carosso A et al	2020	Pre-labor anorectal swab for SARS-CoV-2 in COVID-19 pregnant patients: is it time to think about it?	https://www.ejog.org/article/S0301-2115(20)30202-5/pdf
Hu X et al	2020	Severe Acute Respiratory Syndrome Coronavirus 2 (SARS-CoV-2) Vertical Transmission in Neonates Born to Mothers With Coronavirus Disease 2019 (COVID-19) Pneumonia.	https://journals.lww.com/greenjournal/Citation/9000/Severe_Acute_Respiratory_Syndrome_Coronavirus_2.97384.aspx DOI: 10.1097/AOG.0000000000003926
Diaz SA et al	2020	Neonatal first case of SARS-CoV-2 in Spain First case of neonatal infection due to SARS-CoV-2 in Spain.	https://www.sciencedirect.com/science/article/pii/S1695403320301302?via%3Dihub
Zamaniyan M et al	2020	Preterm delivery in pregnant woman with critical COVID-19 pneumonia and vertical transmission.	https://obgyn.onlinelibrary.wiley.com/doi/epdf/10.1002/pd.5713
Hantoushzadeh S et al	2020	Maternal Death Due to COVID-19 Disease.	https://www.ajog.org/article/S0002-9378(20)30516-0/pdf
Schoenmakers S et al	2020	SARS-CoV-2 placental infection and inflammation leading to fetal distress and neonatal multi-organ failure in an asymptomatic woman.	https://doi.org/10.1101/2020.06.08.20110437
Hosier H et al	2020	SARS–CoV-2 infection of the placenta	https://doi.org/10.1172/JCI139569
Shanes ED	2020	Placental pathology in COVID.	https://doi.org/10.1093/ajcp/aqaa089
Kirtsman M et al	2020	Probable congenital SARS-CoV-2 infection in a neonate born to a woman with active SARS-CoV-2 infection.	doi: 10.1503/cmaj.200821
